# Current Role of Delta Radiomics in Head and Neck Oncology

**DOI:** 10.3390/ijms24032214

**Published:** 2023-01-22

**Authors:** David C. Marcu, Cristian Grava, Loredana G. Marcu

**Affiliations:** 1 Faculty of Electrical Engineering & Information Technology, University of Oradea, 410087 Oradea, Romania; 2 Faculty of Informatics & Science, University of Oradea, 410087 Oradea, Romania; 3 Cancer Research Institute, University of South Australia, Adelaide, SA 5001, Australia

**Keywords:** radiomics, sequential imaging, radiotherapy, chemotherapy, adaptive treatment, outcome prediction

## Abstract

The latest developments in the management of head and neck cancer show an increasing trend in the implementation of novel approaches using artificial intelligence for better patient stratification and treatment-related risk evaluation. Radiomics, or the extraction of data from various imaging modalities, is a tool often used to evaluate specific features related to the tumour or normal tissue that are not identifiable by the naked eye and which can add value to existing clinical data. Furthermore, the assessment of feature variations from one time point to another based on subsequent images, known as delta radiomics, was shown to have even higher value for treatment-outcome prediction or patient stratification into risk categories. The information gathered from delta radiomics can, further, be used for decision making regarding treatment adaptation or other interventions found to be beneficial to the patient. The aim of this work is to collate the existing studies on delta radiomics in head and neck cancer and evaluate its role in tumour response and normal-tissue toxicity predictions alike. Moreover, this work also highlights the role of holomics, which brings under the same umbrella clinical and radiomic features, for a more complex patient characterization and treatment optimisation.

## 1. Introduction

The clinical management of head and neck carcinomas (HNC) involves a number of challenges, both regarding tumour control and normal tissue toxicity owing to the particularities of head and neck anatomy and radiobiology. Resistance to radio- and/or chemotherapy are common factors leading to treatment failure or loco-regional recurrence [1,2]. There are some key tumour characteristics, including hypoxia, proliferative ability, the fraction of cancer stem cells, intrinsic radio-resistance, as well as the human papillomavirus (HPV) status, which should be considered when treating HNC patients. Biomarkers for the identification of the above tumour properties are available and can assist with patient stratification to increase tumour control [3]. The detection of genes responsible for chemo-resistance via pharmacogenetics has been gaining more attention recently, to better personalise chemotherapy as a function of the patient’s genetic makeup [4].

Another important aspect of head and neck oncology concerns the preservation of healthy structures. Given the anatomically challenging tumour sites in this patient group, the surrounding normal tissue is often severely affected, particularly the salivary glands which develop radiation-induced xerostomia. Therefore, monitoring both tumour response and treatment-related side effects are critical factors impacting the therapeutic index and patients’ quality of life.

Advances in molecular and functional imaging in head and neck oncology facilitate more accurate diagnoses and therapeutic recommendations, which are, further, applied for treatment-response monitoring and adaptation. Lately, the implementation of artificial intelligence (AI) in medical imaging allows for the identification of highly detailed and robust tumour-imaging features, providing radiomics—the quantitative approach to medical images—with the status of a strategic field in radiology.

Medical images are fundamental tools for the establishment of diagnosis, response to therapy and patient monitoring during and post therapy. Radiomic image analysis is based on the idea that a medical image depends on the phenotype, genotype and molecular characteristics of the imaged region, but some of the information is not observable with the naked eye [5,6]. The advanced mathematical and statistical analysis of the features extracted from the image has greatly enhanced clinical decision making, followed by the employment of AI techniques to further improve accuracy in the detection, classification, image segmentation and prognostication of treatment outcomes. While the predictive power of radiomics in chemo-radiotherapy is still under scrutiny, the employment of radiomics as a prognostic biomarker in head and neck cancer has been proposed [7].

Perhaps a more powerful tool in the evaluation and interpretation of image features is the assessment of changes exhibited by these features over the course of therapy. The change in quantitative features extracted from longitudinal images acquired at different time points along the course of treatment and follow-up is known as delta radiomics. The delta-radiomic analysis, through deep-learning techniques, of images taken at multiple time points enables a more accurate clinical-outcome prediction than single-image radiomic analysis. This comparative analysis of image features during treatment should be embraced particularly for aggressive, radio-resistant tumours, to guide treatment and optimize the outcome. 

The aim of this overview is to examine the current status of delta radiomics in the management of head and neck cancer, assessing its role in both tumour response and normal tissue toxicity predictions. The fact that most studies were published over the past five years highlights the novelty of this field, and holds promise for future developments in the optimisation of head and neck cancer treatment.

## 2. The Facets of Delta Radiomics in Head and Neck Cancer Management

Current applications of delta radiomics in head and neck oncology show various trends, including:

Diagnostic accuracy;
Tumour response evaluation;
Prediction of normal tissue toxicity;
Potential tool for identification of features used for treatment adaptation.

The sections below summarise the published literature to date on delta-radiomic studies, often presented in the literature under the terminology of the *sequential-imaging*-based assessment of tumour/normal tissue effects.

### 2.1. Delta Radiomics in Diagnostic Accuracy

Most studies on delta radiomics fit into one of the aforementioned categories. Nevertheless, the evaluation of sequential image features can be applied for other purposes, such as differentiating between benign and malignant entities in HNC [8]. In their PET/CT-based study retrospectively undertaken on 56 patients with suspected or confirmed head and neck malignancy, Pietrzak et al. employed sequential FDG-PET examinations to compare the fluctuations in metabolic activity over time (60 and 90 min post injection) for staging purposes [8]. Using the standardized uptake value and the retention index as parameters, they showed that sequential FDG-PET/CT scanning increases specificity and provides more accurate information to assist in differentiating between benign and malignant lymph nodes in HNC.

### 2.2. Delta Radiomics in Tumour Response Evaluation

Various imaging techniques were employed for tumour-response evaluation via sequential image analysis or delta radiomics (Table 1). Quantitative evaluation of ultrasound (US) images was undertaken by Tran et al. during the course of radiotherapy in 36 HNC patients as part of a clinical trial (NCT03908684) having as its main objective the identification of US-based parameters that can serve as early predictors of complete or partial response to radiotherapy. Lymph-nodes images were acquired 24 h, 1 week and 4 weeks after the start of radiotherapy [9]. Quantitative US spectral analysis was applied to compute US parameters for texture features. The naïve-Bayes algorithm used for classification was found to be the best predictor of tumour response to treatment for all time points, showing the potential of US delta radiomics for the early assessment of response to radiotherapy, with a prediction rate of 85% at 4 weeks after the start of treatment.

A study originating from the same clinical trial as above (NCT03908684) reported by Fatima et al. aimed to investigate the role of quantitative US during radiotherapy as a predictive biomarker of tumour recurrence in 51 HNC patients with node-positive carcinomas [10]. Quantitative US images acquired at 1 and 4 weeks after the commencement of radiotherapy were assessed for spectral and textural features and compared (delta features). Of three machine-learning classifiers employed for the radiomics model, Fisher’s linear discriminant, k nearest neighbours, and support vector machine, the latter showed the best performance, with an accuracy of 80% at the 1-week time point (using the baseline image as reference) and 82% at the 4-week time point.

One of the advantages of US imaging compared to CT or PET is the lack of patients’ exposure to ionizing radiation, an aspect that receives more attention in today’s oncology in order to minimize the risks of radiation-induced effects. Furthermore, US devices are less expensive and more convenient due to their portable versions, another important factor that should be considered when monitoring HNC patients. The accrual of a larger number of patients in the above studies would strengthen their prediction power and shed more light on the role of US delta radiomics for the early prediction of treatment-response monitoring and/or recurrence, allowing clinicians to intervene with treatment adjustments for a further optimized outcome.

The study by Morgan et al. employed machine-learning techniques in the attempt to stratify patients at risk of treatment failure using delta-radiomic analysis between baseline CT images and cone-beam CT (CBCT) scans taken during therapy [11]. There were at least three sets of images available for each of the 90 patients included in the study: the baseline CT and two CBCTs—one prior to the first dose fraction and one prior to fraction 21. The explainable boosting machine classifier was used as a machine-learning model. The novelty of the study consisted of the development of a fused ensemble model for the parallel analysis of primary and nodal HNC structures within the same patient, enabling a high discriminatory ability to predict early local failure. The most common delta features included in the study were shape features, particularly the change in sphericity and in maximum 3D diameter, which were in direct correlation with tumour shrinkage. While the model needs validation on a larger cohort, these preliminary results could support early decision for treatment adaptation in patients at high risk of local failure [11].

The use of CBCT in image-guided HNC therapy was exploited by others, through the analysis of radiomic signature changes between baseline CBCT and subsequent CBCT images acquired during treatment [12]. The study aimed to gather longitudinal information of radiomic features and to evaluate treatment-induced changes in these features for the prediction of outcome in combination with clinical factors. Patients having at least four CBCT image acquisitions (including the baseline) were considered eligible for the study. Single time-point feature selection was conducted based on the receiver operating characteristic (ROC) curves, conditioned by an AUC > 0.65. For the longitudinal features selection, the 95% confidence interval was determined for the smallest detectable change, with relevant features being considered those that underwent a detectable change during therapy for at least 5% of patients [12]. Of the three developed models (clinical-based, radiomics-based and combined), the combined model showed the highest accuracy in identifying poor responders. The coarseness (measure of the difference between the central voxel and its neighbourhood) was identified as the most significant radiomic parameter undergoing longitudinal change, while among clinical parameters the change in haemoglobin levels correlated the best with outcome. Radiomic features extracted from the 4th-week CBCT already showed prognostic power for treatment response.

Next to ultrasound and CBCT-based delta radiomics, the role of MRI features was also investigated in radiomics settings. Xi et al. performed pre-treatment MRI radiomics on a large patient lot, with the aim to extract the most optimal features for treatment-response prediction and to compare the radiomics model with the delta radiomics based on MRI images (sequential MRI of axial-fat-suppressed T2-weighted image (FS T2WI) followed by axial-fat-suppressed contrast-enhanced T1-weighted image (FS CE-T1WI)) acquired within 2 weeks before and after chemo-radiotherapy [13]. Both the single time point (radiomics) (AUC = 0.865) and delta-radiomics model (AUC = 0.941) showed good predictive power for tumour response to chemo-radiotherapy in nasopharyngeal cancer patients using MR imaging, potentially allowing for early treatment adaptation and optimisation. 

Another multi-parametric MRI study undertaken on 50 patients with sinonasal cancers investigated the value for treatment outcome prediction after induction chemotherapy. The investigation included both mono-modality delta-radiomics signatures as well as fused signature for T1-weighted, T2-weighted, and apparent diffusion coefficient (ADC) maps [14]. The addition of ADC map information to either T1- or T2-weighted features improved the AUC values, confirming the importance of ADC maps for the predictive model. The study showed that early prediction of response to induction chemotherapy in this patient group using radiomics signature is superior to RECIST (Response Evaluation Criteria in Solid Tumours)-based radiological predictions. The clinical use of the radiomic model leads to the possibility of early treatment adjustments for non-responsive patients after induction chemotherapy, avoiding unnecessary toxicities. The conclusions of this study are in line with similar reports showing that delta-radiomics models are preferred to single time-point models in predicting tumour response to therapy [14]. 

### 2.3. Delta Radiomics in Normal Tissue Toxicity Evaluation

Patients undergoing radiotherapy for HNC often develop severe and debilitating side effects. One of the most common normal tissue toxicities affects the functionality of the parotid glands, leading to xerostomia. Therefore, it is not surprising that most studies involving delta radiomics developed such models to analyse their predictive power for acute or late xerostomia, allowing for interventions during treatment to reduce the magnitude of side effects (Table 2). 

Van Dijk et al. aimed to identify a biomarker for late xerostomia (12 months post radiotherapy) based on CT imaging data acquired after treatment completion [15,16]. This was achieved in a cohort of 107 HNC patients by quantifying the differences in parotid-gland surface reduction before and 6 weeks after radiotherapy. The geometric difference between the contralateral parotid gland surface was shown to be the most predictive factor of late xerostomia (AUC = 0.90). A non-linear relationship was found between the mean dose received by the parotids and the change in the parotids’ surface, with an increase in the parotid surface reduction with increasing doses (up to 40 Gy); however, at higher doses, the reduction in gland surface decreased. This observation suggests different reactions of the parotid to higher doses owing to necrosis-caused inflammatory enlargement, rather than apoptotic cell death [15]. A sister study enrolling 68 patients was conducted by the same group with similar goals, though with a different delta-radiomic approach, based on pre-treatment and weekly CT images acquired during radiotherapy [16]. The main features extracted from the CT images were intensity, texture and geometric characteristics (gland surface) of the parotid glands. The most significant delta feature was the change in the parotid surface, which is associated with late xerostomia (12 months post-therapy). While this correlation was achieved for CT delta radiomics applied for all treatment weeks (*p* < 0.04), the highest significance, with the largest regression coefficient, was achieved in week 3 (*p* < 0.001). Therefore, the mid-treatment CT assessment of parotid surface changes as compared to baseline CT (pre-treatment) could potentially identify patients at risk of developing late xerostomia, allowing for timely treatment adaptation. While the results are highly promising, they are required to be externally validated before clinical implementation.

Daily CT images were employed for delta-radiomics analysis of 59 HNC patients in order to identify possible correlations between the severity of acute xerostomia and changes in CT-histogram texture features [17]. Among all parameters investigated, the changes in mean CT number and in the parotid volume were correlated with xerostomia grades when combined in the same predictive model (r = 0.71, *p* < 0.00001). The highest precision of acute xerostomia severity was predicted by the 5th-week delta radiomics. In a study conducted on 35 nasopharyngeal cancer patients, the changes in the amount of saliva were found to be an important predictor of acute xerostomia, next to changes in normalized feature values assessed on CT images between fraction 0 and fraction 10 of radiotherapy [19]. 

The risk of chronic xerostomia after HNC radiotherapy was evaluated using CBCT-based delta radiomics in a retrospective study of 119 patients [18]. Delta radiomics consisted of average weekly changes in the assessment of mean Hounsfield unit intensity and parotid volume, using week-1 CBCT images as baseline. A significant correlation was found between mid-treatment volume change and mean parotid dose. The predictive value of the radiomics model was compared with clinical and dose-volume histogram models by means of AUC. The delta-radiomics model showed slightly higher prediction value for grade-1 xerostomia as compared to the clinical model (AUC = 0.719 vs. 0.709), while the addition of delta-radiomic features (changes in contralateral parotid volume) to the clinical model improved the predictive performance for higher grade toxicities from AUC = 0.692 to 0.776 [18].

Another study that aimed to compare the statistical power of delta-radiomics model to predict xerostomia compared to dose-volume parameters (clinical model) analysed radiomics data and their variations from daily megavoltage CT images from 117 HNC patients [20]. Early- and mid-treatment radiomics data were found to be the most predictive for xerostomia symptoms at 6 months. The conclusion of this work is in line with that of Rosen et al., showing a better model performance when radiomics (textural) features are added to traditional clinical data.

An interesting study looking at the temporal evolution of radiomic features rather than at changes between different time points (delta radiomics) was recently reported by Barua et al. [21]. The focus of the study was the risk evaluation of osteoradionecrosis in the mandibular bone of oropharyngeal cancer patients, using temporal trajectories of radiomic features that were derived from serial contrast-enhanced CT images acquired at three different time points: pre-treatment, 2 months, and 6 months post-radiotherapy. Their aim was to develop a predictive model using multivariate functional principal component analysis to assess temporal (kinetic) CT changes in mandibular subvolumes of patient at high risk for osteoradionecrosis. AUC-based model evaluation showed superiority over the radiomic kinetics model when compared to clinical or even delta radiomics, opening new avenues for image analysis through novel statistical approaches [21].

### 2.4. Delta Radiomics as a Potential Tool for Treatment Adaptation

Imaging plays an essential role in monitoring the treatment response of oncological patients. While the evaluation of post-treatment and follow-up images offer important information regarding treatment success, image analysis during the course of therapy can potentially assist with treatment adaptation, thus contributing to a more customized therapy. A number of recent studies using sequential PET/CT images acquired during treatment confirmed that changes in tumour dynamics based on hybrid image-feature variations call for treatment adjustments to improve patient outcome [22,23]. 

Given that tumour hypoxia is associated with resistance to therapy and cancer recurrence, Lazzeroni et al. investigated the association between the dynamic nature of hypoxia during chemo-radiotherapy in head and neck cancer patients and outcome prediction by analysing sequential PET/CT image features. The study employed ^18^FMISO (^18^F-fluoromisonidazole) as an imaging agent, which is a PET radiotracer with selective uptake in hypoxic cells. Oxygen partial-pressure maps were then evaluated and compared through the progression and severity of hypoxic sub-volumes within the tumour, which revealed good correlations between the hypoxic areas and treatment outcome. PET/CT image features derived from the first two weeks of chemo-radiotherapy demonstrated the predictive power of delta radiomics in radio-resistant head and neck cancer patients [23]. Another study undertaken in head and neck cancer patients with hypoxic tumours, in line with the above-presented findings, reported that radiomic features of hypoxia-specific PET/CT images, but also variations in these features during chemo-radiotherapy, predict survival in this patient group. The study revealed that a higher homogeneity of tumour hypoxia during therapy is associated with a better treatment outcome [22]. 

Tumour proliferation during therapy is another common feature of HNC which can hinder treatment success. In view of this, ^18^F-FLT PET (3′-deoxy-3′-(18)F-fluorothymidine), a proliferation-specific tracer, was employed to monitor early tumour response to treatment and to identify possible correlations between PET parameters and outcome [24]. The study involved 48 HNC patients who underwent sequential ^18^F-FLT PET scans before and during the 2nd and 4th weeks of radio/chemotherapy. A decline in SUVmax higher than 45%, and of the PET-segmented gross tumour volumes using visual delineation (GTVVIS) greater than the median, during the first 2 weeks of therapy correlated with superior 3-year disease-free survival. A further decrease in the GTVVIS in the 4th week of treatment also correlated with better 3-year loco-regional control (100% vs. 68%, *p* = 0.021), showing that a change in ^18^F-FLT uptake early during treatment is a strong predictor of clinical outcome and could serve as a biomarker for treatment personalisation and adaptation [24,25].

An important aspect that often needs intervention and adaptation of the treatment plan in HNC radiotherapy is tumour volume alteration due to weight loss, tumour shrinkage or variations in tumour position and shape [26]. Changes in tumour volume over the course of therapy impact not only the tumour dosage but also on the surrounding healthy organs that could receive an overdose, leading to side effects. To predict early volumetric changes, Illiadou et al. developed a delta-radiomic model based on weekly CBCT images in a cohort of 40 HNC patients, focusing on parameters related to the clinical target volume and the parotid glands [27]. A recursive-feature elimination with correlation bias (RFE-CBR) feature-selection procedure combined with support vector machine (SVM) classifiers was employed to predict anatomical changes in the initial tumour volume. A 0.90 prediction accuracy was achieved (AUD = 0.91) with the selected radiomic features (13 features for the tumour volume and 6 for the parotids). Delta radiomics of weekly CBCT images during HNC radiotherapy using week 1 CBCT as a baseline was shown to provide important information on volume changes from the first week of therapy, which could identify the need for and guide treatment adaptation.

## 3. Holomic Approach in Head and Neck Oncology towards Personalised Therapy

A complex characterization of patient- and tumour-related landscapes requires a holistic methodology which includes both imaging (radiomics) as well as non-imaging data. While placing radiological imaging at the centre of diagnostics and treatment monitoring, supplemental information such as blood markers, immunohistochemistry data, patient characteristics (age, body mass index, lifestyle-derived factors) constitute satellite data which enriches the radiomics information extracted purely from imaging. This ensemble of data represents a more rounded characterization of the patient, used as input for artificial-intelligence models for classification and prediction.

**Figure 1:**
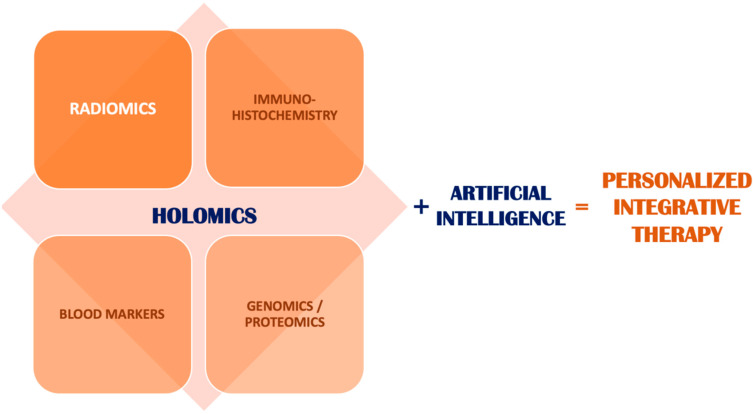
Schematic illustration of the equation of personalised therapy in oncology.

This holistic multi-omics field, which encompasses both radiomics and non-imaging data to define patient characteristics, is referred to as holomics. The holomic approach does not require major structural changes in the use of AI within the management of HNC. Feeding the holomic data into AI models which were modified to accept these inputs will generate better patient-characterization outputs than radiomics analysis alone, taking us closer to personalized integrative therapy (Figure 1) [28].

The potential of survival prediction using clinical parameters combined with multi-imaging radiomics based on MRI and ^18^F-FDG-PET was examined by Martens et al. on a cohort of 70 HNC patients. The aim of the study was to evaluate the correlations between a set of MRI parameters (diffusion-weighted, intravoxel incoherent motion, dynamic contrast-enhanced MRI) and PET/CT features regarding tumour characteristics and to further assess their predictive value of various clinical endpoints when combined with clinical information (i.e., satellite features) in patients undergoing chemo-radiotherapy [29]. Parameters such as HPV status, tumour volume, permeability, and extravascular extracellular space on dynamic contrast-enhanced MRI were predictive for loco-regional recurrence and overall survival. Other combined imaging parameters added complementary value to tumour-feature analysis, showing that cellularity on the apparent diffusion coefficient map (MRI) and the metabolic rates (standardized uptake value, PET) offered additional predictive value for overall survival.

The study reported by Sellami et al. [12] (Table 1) analysed the combined radiomic (CBCT-based) features with clinical characteristics undertaking delta-radiomic analysis of the combined model. Of the clinical characteristics, the following were included: age, gender, performance status, smoking habit, tumour stage, anatomic location, body mass index, haemoglobin value, radiation dose, concomitant chemotherapy, and treatment interruption. The study revealed that the radiomic feature with the largest change among consecutive CBCT images is coarseness, while the haemoglobin level was the most representative clinical parameter that correlated with outcome [12]. This addition of a clinical parameter to delta-radiomic analysis is another example of a delta-holomic approach towards treatment-outcome prediction.

Rosen et al. demonstrated the added value of clinical data to delta radiomics in predicting chronic xerostomia in HNC patients undergoing radiotherapy [18]. Next to radiomics features, clinical data extracted from dose-volume histograms increase the predictive power of the delta-radiomics model, encouraging the use of combined data for a holomic approach to treatment.

## 4. Challenges and Prospects of Delta Radiomics

There are limitations to delta radiomics. Radiomic features are strongly dependent on image acquisition and reconstruction settings, causing large differences between datasets [30,31]. Furthermore, differences in image processing and segmentation-method variability in textural analysis are other causes of biased data interpretation [32]. The field of radiomics is still lacking standardization; thus, harmonization guidelines are a fundamental requirement for further progress [33,34,35,36]. In a recent work, Welch et al. established a set of safeguards aimed to support radiomic models through a comprehensive analysis of the radiomic signature [37]. Furthermore, non-standardized image parameters [38] and differences in fusion algorithms for hybrid imaging [39] make it challenging to compile a standardized dataset.

One non-technical but, nevertheless, important challenge in implementing delta radiomics is patient adherence to imaging timelines. Patient non-compliance with follow-up protocols can lead to a lack of the necessary information for adequate delta-radiomic analysis [40].

While radiomics features provide useful information from diagnostic through treatment and follow-up, the definition of exact values for these features is difficult due to inter-patient differences in radiomic signatures. Delta radiomics provides a valuable service in allowing the analysis of data for one particular patient in time, eliminating inter-patient variability. The possibility of following a set of features for the particular patient over time is a critical step in achieving true personalized treatment. 

## Figures and Tables

**Table 1:** Compilation of delta radiomics studies for evaluation/prediction of tumour response to therapy.

Study [Ref]	Aim of Study	Imaging Technique for Delta Radiomics	Outcome
Tran et al., 2020 [9] 36 HNC patients	Treatment-response monitoring	Quantitative ultrasound (spectral and texture parameters)	The best prediction accuracy was offered by single-feature naïve-Bayes classification (80% at 24 h; 86% at 1 week and 85% at 4 weeks after commencement of RT).
Fatima et al., 2021 [10] 51 HNC patients	Prediction of recurrence	Quantitative ultrasound (spectral and texture parameters)	The support vector machine classifier showed the best performance using delta radiomics in terms of accuracy (80% at week 1 and 82% at week 4) and AUC (0.75 at week 1 and 0.81 at week 4).
Morgan et al., 2021 [11] 90 HNC patients	Prediction of local failure	CT and intra-treatment CBCT	The highest (AUC = 0.871) at predicting local failure was achieved by the fused ensemble model. The same model scored the highest (AUC = 0.910) at predicting local failure for HN nodes.
Sellami et al., 2022 [12] 93 HNC patients	Prediction of response to radiotherapy	CBCT	Coarseness was the most significant radiomic feature, while haemoglobin level was most significant for the clinically relevant features. The combined clinical + radiomic model achieved AUD = 0.99 for treatment-response prediction.
Xi et al., 2022 [13] 272 HNC patients (nasopharynx)	Prediction of response to induction chemo + chemoradiotherapy	Multi-parametric MRI	LASSO-based feature selection was conducted: seven feature subsets were identified for the pre-treatment MRI radiomic model and 12 subsets for the delta-radiomics model. Both models were able to predict tumour response to therapy.
Corino et al., 2022 [14] 50 HNC patients (sinonasal)	Prediction of response to induction chemotherapy	Multi-parametric MRI	Three mono-modality delta-radiomics signatures determined for T1-weighted (AUC = 0.79), T2-weighted (AUC = 0.76) and apparent diffusion coefficient maps (AUC = 0.93). Fused signature for all features was 0.89.

Abbreviations: MRI = magnetic resonance imaging; CT = computed tomography; FDG-PET = fluorodeoxyglucose-positron emission tomography; CBCT = cone beam computed tomography; RT = radiotherapy; AUC = area under the curve; SUV = standardized uptake value.

**Table 2:** Compilation of delta-radiomics studies for normal tissue toxicity evaluation.

Study [Ref]	Aim of Study	Imaging Technique for Delta Radiomics	Outcome
van Dijk et al., 2017, 2019 [15,16] 107 HNC patients (2017) 68 HNC patients (2019)	Prediction of radiation-induced late xerostomia	CT	The most predictive feature was the change in the contralateral parotid-gland surface, which showed significant correlation with late xerostomia.
Wu et al., 2018 [17] 59 HNC patients	Early prediction of acute xerostomia during RT	CT	Combined changes in CT histogram features (mean CT number, parotid volume) correlated with acute xerostomia.
Rosen et al., 2018 [18] 119 HNC patients	Prediction of chronic xerostomia after RT	CBCT	The addition of delta radiomics to doses/clinical models improves the prediction of chronic xerostomia.
Liu Y. et al., 2019 [19] 35 HNC patients (nasopharynx)	Early prediction of acute xerostomia during RT	CT	Saliva amount changes during radiotherapy and NFV as well as changes in NFV between fractions 0 and 10 of RT provide the best prediction of acute xerostomia.
Berger et al., 2022 [20] 117 HNC patients	Prediction of radiation-induced xerostomia	CT	Delta-radiomics model outperformed the clinical model at predicting xerostomia at 6-, 12- and 24-months post-radiotherapy.

Abbreviations: CT = computed tomography; CBCT = cone-beam computed tomography; RT = radiotherapy; AUC = area under the curve; NFV = normalized feature values.

## Data Availability

All data used in this review is available from the reference list.
